# Prevalence and risk factors for foot and ankle musculoskeletal disorders experienced by nurses

**DOI:** 10.1186/1471-2474-15-196

**Published:** 2014-06-05

**Authors:** Lloyd F Reed, Diana Battistutta, Jeanine Young, Beth Newman

**Affiliations:** 1 School of Clinical Sciences, Queensland University of Technology, Brisbane, Queensland, Australia; 2 Institute of Health and Biomedical Innovation, Queensland University of Technology, Brisbane, Queensland, Australia; 3 School of Nursing and Midwifery, University of the Sunshine Coast, Sunshine Coast, Queensland, Australia; 4 Visiting Professor, Children’s Health Queensland Hospital and Health Services, Brisbane, Queensland, Australia; 5 School of Public Health and Social Work, Queensland University of Technology, Brisbane, Queensland, Australia

## Abstract

**Background:**

Nurses are at high risk of musculoskeletal disorders (MSDs). Although the prevalence of MSDs of the lower back, upper limbs, neck and shoulders have been reported previously in nursing, few studies have evaluated MSDs of the foot and ankle. This study evaluated the prevalence of foot and ankle MSDs in nurses and their relation to individual and workplace risk factors.

**Methods:**

A self-administered survey incorporating the Nordic Musculoskeletal Questionnaire (NMQ) was distributed, over a nine-week period, to all eligible nurses (n = 416) working in a paediatric hospital in Brisbane, Australia. The prevalence of MSDs for each of the NMQ body regions was determined. Bivariate and multivariable logistic regression analyses were conducted to examine the relationships between activity-limiting foot/ankle MSDs and risk factors related to the individual (age, body mass index, number of existing foot conditions, smoking history, general physical health [SF36 Physical Component Scale], footwear features) or the workplace (level of nursing position, work location, average hours worked, hours worked in previous week, time since last break from work).

**Results:**

A 73% response rate was achieved with 304 nurses completing surveys, of whom 276 were females (91%). Mean age of the nurses was 37 years (±10), younger than the state average of 43 years. Foot/ankle MSDs were the most prevalent conditions experienced by nurses during the preceding seven days (43.8%, 95% CI 38.2-49.4%), the second most prevalent MSDs to impair physical activity (16.7%, 95% CI 13.0-21.3%), and the third most prevalent MSD, after lower-back and neck problems, during the preceding 12 months (55.3%, 95% CI 49.6-60.7%). Of the nurse and work characteristics investigated, obesity, poor general physical health, existing foot conditions and working in the intensive care unit emerged as statistically significant (p < 0.05) independent risk factors for activity-limiting foot/ankle MSDs.

**Conclusions:**

Foot/ankle MSDs are common in paediatric hospital nurses and resulted in physical activity limitations in one out of every six nurses. We recommend targeted education programs regarding the prevention, self-management and treatment strategies for foot/ankle MSDs. Further research is needed into the impact of work location and extended shift durations on foot/ankle MSDs.

## Background

Nurses are at high risk of work-related musculoskeletal disorders (MSDs) with lower-back pain/discomfort being the most frequent and past-year prevalence estimates ranging from 32% [1] to 90% [2]. Prevalence of MSDs at other sites, including the neck (12% [1] to 52% [3]), shoulders (17% [1] to 48% [4]) and knees (7% [1] to 68% [4]), are somewhat lower.

Daraiseh et al. reviewed a number of studies from the 1990s, investigating prevalence of MSDs in nurses and reported that foot problem prevalence ranged from 3.7 to 40% [5]. A small number of studies since then [1,4,6-8] have reported the prevalence of foot/ankle MSDs ranging from 1.8% [8] to 74% [9]. This disparity reflects differences in defining foot problems, diversity between the characteristics of the nurses studied (student nurses to experienced practitioners, young vs middle aged), differences in their workplaces (hospital and community) and differences in the sociocultural environments and health systems (Africa, Iran, Japan, Taiwan, Europe, United States). In these studies, relationships between potential individual and workplace risk factors and MSDs affecting the foot/ankle have not been investigated in detail, although one Brazillian study [6] compared the percentage of nurses with foot MSDs across groups defined by personal, work and demographic characteristics. They reported a higher annual prevalence of foot MSDs in nurses who were obese compared to those of normal bodyweight (44.9% vs 23.9%) and for nurses on the lowest annual income compared to those in the highest income bracket (36.5% vs 21.2%) [6]. Little is known about the prevalence and risk factors for foot/ankle MSDs in the nursing workforce in Australia, particularly for nurses working in paediatric environments, which is why our study was conducted in Brisbane, Australia.

## Methods

A cross-sectional study was conducted to establish the prevalence and risk factors for foot/ankle MSDs experienced by a cohort of hospital nurses.

### Study population

The study took place in a large paediatric hospital in Brisbane, Australia. Ethical approval was granted by Queensland University of Technology Human Research Ethics Committee and subsequent approval was given by the relevant hospital ethics committee. Letters of support and approval were provided by the occupational health and safety manager and nurse unit managers of the participating hospital. Questionnaires were distributed to all temporary and permanent nursing staff rostered to work at the time of the study (n = 416) in consultation with Nurse Unit Managers. All nurses from participating wards/work areas were provided with the questionnaire via the internal mail system. Consent to participate was implied by the return of a completed questionnaire. Two waves of survey distributions, followed by a non-responder survey, were used over a nine week period to promote participation, consistent with a modified Dillman technique [10].

### Questionnaire development and sample size estimation

A 13-page questionnaire was developed for the study, using a seven-step process [11] that included obtaining input from nurses, podiatrists, occupational health practitioners and epidemiologists. The survey was piloted with researchers, nurses and health care workers (n = 25), and revised to improve clarity and speed of completion. The final questionnaire comprised four parts containing questions about: 1) personal demographic and work characteristics; 2) MSDs (incorporating the standardised Nordic Musculoskeletal Questionnaire (NMQ) [12]); 3) foot health and foot conditions; and 4) general health status (SF36 Acute Version) [13]. An open-ended question “*Is there anything else you would like to tell us about your foot problems or musculoskeletal problems to help us to better understand these issues?*” was included as the final question in the survey. An *a priori* calculation of sample size assuming the prevalence of foot problems was 50%, determined that 385 responses would be required to ensure a 5% precision for the prevalence estimate. Lower prevalences would require fewer responses for the same precision. Previous staff surveys at the hospital suggested that more than 200 responses would be received. A final sample size target of all eligible permanent and temporary staff nurses rostered to work in the hospital during the recruitment period was used (n = 416).

### Explanatory variables

Proposed risk factors for foot/ankle MSDs were determined based on the literature and discussions with nurses and podiatrists during the questionnaire development phase, focusing on individual characteristics and workplace characteristics. The individual characteristics included age, BMI, smoking history, general physical health (SF36 Acute- Physical Component Scores (PCS) [13]), number of self-reported foot conditions associated with foot discomfort (bunions, curled toes, flat feet, high arches, corns or callous, heel spur), and footwear features (shoe style, heel heights). The number of foot conditions was summed and categorised (none, one, two or more) to give a crude index of foot conditions. The workplace characteristics included: level of nursing position, hospital location, (mean) hours worked per week, hours worked in the last week and length of time since last break from work.

### Outcome variables

The three ankle/foot questions from the NMQ [12] component of the questionnaire were: ‘trouble (such as ache, pain, discomfort, numbness)’ in the last 12 months, ‘trouble during the last seven days’ and ‘prevented from carrying out normal activities (e.g. job, housework, hobbies) because of this trouble’ in the last 12 months. The outcome variables based on these questions were foot/ankle MSDs in the last 12 months, in the last seven days and activity-limiting foot/ankle MSDs. Reporting prevalence estimates for all three variables was deemed appropriate, given the small number of studies in this area have generally reported the annual prevalence in isolation.

### Data analysis

The data were double-entered into a data set and imported into the Statistical Package for Social Sciences version 12.0 (SPSS Inc. – Chicago IL) which was used for all subsequent statistical analyses. SF36 PCS scores were calculated using the recommended formulae [13] and substituting Australian population norms [14]. Ambiguous responses and missing data for the SF36 were dealt with using techniques described by Ware and colleagues [13].

Continuous explanatory variables were coded into categorical variables using objective definitions (e.g. quartiles for SF 36 PCS scores) and with reference to categories accepted in the literature (e.g., World Health Organisation BMI groups [15]). This allowed for group comparisons and easier descriptions of differences between subjects based on these characteristics. *A priori* criteria were adopted to define meaningful relationships between adverse risk factors and outcome variables (statistically significant p < 0.05 or odds ratio > 2.0) so that neither statistical significance nor effect size dominated interpretation of potential importance, in this situation where sample size was calculated to ensure precision of MSD prevalence, rather than to power particular testing of hypotheses around risk factors.

Reliability testing of the NMQ responses from cashier workers (n = 44) has shown non-identical responses varying from 7-26% for annual prevalence and 6-19% for weekly prevalence questions after a one week test-re-test period. Non-identical responses for annual disability were lower, ranging from 0-8% [16]. Reliability of an extended version of the NMQ completed by 59 nursing students, after a 24 hour test-retest period, was better, with non identical responses of 10% for annual prevalence and 3% for annual disability on the foot problem questions [17].

Bivariate associations between the proposed risk factors and the specific outcome variable of activity-limiting foot or ankle MSDs were considered using logistic regression modelling. A multivariable model was used to assess the relationships between risk factors and the presence of activity-limiting foot or ankle MSDs, as these were considered to be the MSDs that had the greatest impact on the nurses, as well as being the most reliable based on the literature. For this model, the variable ‘average hours per week’ was collapsed to full-time and less than full-time workload (36 or more hours per week, <36 hours per week) because of the relative homogeneity in odds ratios across the categories used in the bivariate analyses. Odds ratios were reported in comparison to the designated referent (lowest risk group).

## Results

### Sample characteristics

Of the 416 eligible nurses, 304 (73% response rate) returned a completed survey used for analysis. Twenty-three participants were males (7.6%), 276 were female (92.4%), 5 did not indicate gender. The ratio of male to female nurses followed the state nursing workforce average (9%: 91%) [18], as did the distribution of nurses across job levels and the proportion of nurses working full-time (53.9%) and part-time (46.1%) [19] and closely reflected the nursing workforce in Queensland in 2011 (9.8% males, 49.5% part-time) [20]. However, the mean age was 37 years, which was younger than the state average of 43 years [18] and lower than the 2011 national mean age of 44.2 yrs [20]. Twenty-six nurses (24% of the non-responders) returned a one-page non-respondents’ survey. Table 1 presents summary statistics for key characteristics of the responders and non-responders, with minimal differences between the groups. Compared to responders, the prevalence of foot MSDs in the last 12 months was 9% greater for non-responders (64%) but was essentially the same for foot MSDs in the last 7 days (40%) and for activity-limiting foot MSDs (16%).

**Table 1:** Summary statistics of nurses completing cross-sectional survey (n = 304)

	**MSD survey responders n = 304**		**Non-responders survey n = 26**	
**Characteristic**	**Mean**	**Std dev**	**Mean**	**Std dev**
Age (years)	37.1	10.2	32.6	9.0
Height (cm)	165.7	8.2	164.9	7.8
Weight (kg)	69.0	13.9	66.7	13.1
BMI^1^ (kg/m^2^)	25.2	4.7	24.5	4.6

^1^Body Mass Index.

### Prevalence of foot and ankle MSDs compared to other MSDs

**Figure 1:**
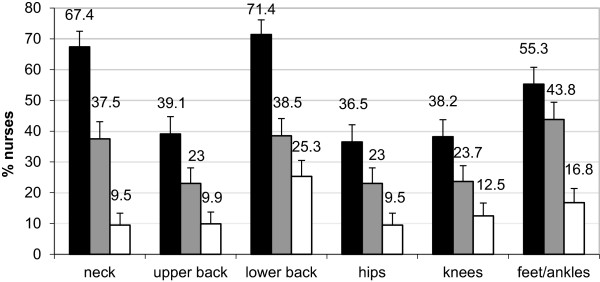
Prevalence of MSDs reported for each body region (Black shaded) problem in the last 12 months, (Grey shaded) problem in the last 7 days, (White shaded) problem in the last 12 months that limited normal activity.

The prevalence of MSDs across the different body regions are presented in Figure 1. Foot/ankle MSDs were the most prevalent conditions experienced by nurses during the preceding seven days (43.8%, 95% CI 38.2-49.4%), the second most prevalent MSD to impair nurses’ physical activity (16.7%, 95% CI 13.0-21.3%) and the third most prevalent MSD, after lower-back and neck problems, to be experienced by nurses during the preceding 12 months (55.3%, 95% CI 49.6-60.7%).

### Relationships between foot or ankle MSDs and proposed risk factors

The bivariate relationships between the nurse characteristics and activity-limiting foot/ankle MSDs can be seen in Table 2. The nurse characteristics of older age (50+), obesity, general physical health (PCS) below the median and having 2 or more existing foot conditions more than doubled the odds of experiencing activity-limiting foot/ankle MSDs and each of these associations was statistically significant. There was a threefold increase in the odds of activity limiting foot/ankle MSDs for the work characteristic of working more than 32 hours per week, although this was not statistically significant and the odds more than doubled for nurses working in ICU (Table 3). Nurses who reported using insoles/orthoses in their footwear were also more likely to report disabling foot conditions (Bivariate analyses).

**Table 2:** Bivariate analyses of individual risk factors for activity-limiting foot/ankle MSDs among nurses

**Risk factor**	**n**	**%**	**OR**	**CI (95%)**	**Sig.**
*Age (years)*					
<30	75	27.4	Referent		0.11
30-39	100	36.5	1.28	(0.56, 2.89)	0.56
40-49	66	24.1	0.69	(0.25, 1.90)	0.47
50+	33	12.0	2.53	(0.95, 6.74)	0.06
*BMI*^*1*^*(kg/m*^*2*^*)*					
Underweight/normal	139	53.3	Referent		<0.001^*^
Overweight	79	30.3	1.63	(0.73, 3.63)	0.23
Obese/morbidly obese	43	16.5	4.43	(1.94, 10.10)	<0.001^*^
*Smoking history*					
Never smoker	180	62.5	Referent		0.46
Previous smoker	85	29.5	0.95	(0.49, 1.87)	0.89
Current smoker	23	8.0	0.42	(0.09, 1.90)	0.26
*SF-36 PCS*^*2*^					
Quartile 4 (>55.96)	70	25.0	Referent		<0.001^*^
Quartile 3 (51.96-55.95)	69	24.6	1.29	(0.33, 5.02)	0.71
Quartile 2 (48–51.95)	71	25.4	3.70	(1.14, 11.97)	<0.001^*^
Quartile 1 (0–47.99)	70	25.0	8.61	(2.80, 26.48)	<0.001^*^
*No. of foot conditions*					
None	104	35.5	Referent		<0.001^*^
One	103	35.2	1.48	(0.85, 2.56)	0.16
2 or more	86	29.4	2.68	(1.48, 4.85)	<0.001^*^
*Shoe style*					
Walking shoe- hard sole	47	16.5	Referent		0.59
Walking shoe- soft sole	138	48.4	0.90	(0.40, 2.03)	0.80
Sports/Men’s Shoe/Other	38	13.3	0.69	(0.23, 2.12)	0.52
Ladies dress shoe	38	13.3	0.44	(0.12, 1.52)	0.19
Clog/Mule	24	8.4	0.53	(0.13, 2.14)	0.37
*Shoe - heel height*					
0-2.5 cm	185	65.1	Referent		0.07
> 2.5 cm	99	34.9	1.77	(0.95, 3.29)	0.07
*Wears insoles/Orthoses*					
No	237	82.6	Referent		<0.001^*^
Yes	50	17.4	5.19	(2.62, 10.28)	<0.001^*^

^1^Body Mass Index categories: underweight/normal (<24.99), overweight (25–29.99), obese (>30.00).

^2^SF36 Physical Component Summary Scale.

*statistically significant.

**Table 3:** Bivariate analyses of work-related risk factors for activity-limiting foot/ankle MSDs

**Risk factor**	**n**	**%**	**OR**	**CI (95%)**	**Sig.**
*Level of nursing position*					
Level 1	212	76.8	Referent		0.49
Level 2,3,4	64	23.2	1.29	(0.64, 2.62)	0.49
*Location*					
Ward/Pool^1^/TASU^2^/other^3^	139	47.3	Referent		0.13
Emergency	36	12.2	1.73	(0.66, 4.56)	0.27
Intensive care	41	13.9	2.63	(1.12, 6.20)	0.03
Theatre/Outpatients/Gastro^4^/DPC^5^	78	26.5	1.85	(0.88, 3.91)	0.11
*Average hours worked per week*					
0-23	40	13.9	Referent		0.14
24-31	40	13.9	1.76	(0.39, 7.93)	0.46
32-39	130	45.3	2.94	(0.84, 10.30)	0.09
40+	77	26.8	3.49	(0.96, 12.74)	0.06
*Hours worked last week*					
0-23	73	26.2	Referent		0.44
24-31	48	17.2	1.87	(0.70, 5.02)	0.21
32-39	81	29.0	1.24	(0.49, 3.13)	0.65
40+	77	27.6	1.87	(0.77, 4.54)	0.17
*Time since last break from work*					
< 2 days	84	30.4	Referent		0.90
2 days	74	26.8	1.04	(0.55, 1.94)	0.91
> 2 days	118	42.8	1.13	(0.65, 1.98)	0.66

^1^Nursing Pool – Nurses allocated to different work areas each shift.

^2^Theatre Admission Supply Unit.

^3^Other – includes administration, Central Sterilising.

^4^Gastroenterology outpatients.

^5^Day Procedures Centre.

Multivariable modelling examined the independent contributions of the risk factors that met criteria for meaningful association with activity-limiting MSDs in the bivariate analyses, see Table 4. After adjustment, reporting activity-limiting foot/ankle MSDs was almost five times more likely for nurses who were obese (OR 4.74 CI 1.70-13.23) or those with 2 or more foot conditions (OR 5.59 CI 1.75,17.81) and six times more likely for nurses with physical health scores in the lowest quartile (OR 6.05 CI 1.64, 22.38). Working in the ICU increased the odds almost fourfold (OR 3.87 CI 1.23, 12.12). All of these results were statistically significant.

**Table 4:** Multivariable analysis of selected risk factors for activity-limiting foot/ankle MSDs among hospital-based nurses in Brisbane, Australia (n = 240)

**Risk factor**	**n**	**%**	**OR**	**CI (95%)**	**Sig.**
*Nurse characteristics*					
*Age (years)*					
<30	66	27.5	Referent		0.79
30-39	90	37.5	0.71	(0.25, 2.04)	0.53
40-49	62	25.8	0.52	(0.14, 1.86)	0.31
50+	22	9.2	0.76	(0.18, 3.27)	0.71
*BMI*^*1*^*(kg/m*^*2*^*)*					
Underweight/normal	128	53.3	Referent		0.01^*^
Overweight	70	29.2	1.46	(0.52, 4.09)	0.47
Obese/morbidly obese	42	17.5	4.74	(1.70, 13.23)	<0.001^*^
*SF-36 PCS*^*2*^					
Quartile 4 (>55.96)	63	26.3	Referent		0.02^*^
Quartile 3 (51.96-55.95)	60	25.0	1.21	(0.28, 5.2)	0.80
Quartile 2 (48–51.95)	60	25.0	2.76	(0.76, 9.98)	0.12
Quartile 1 (0–47.99)	57	23.8	6.05	(1.64, 22.38)	0.01^*^
Foot condition - Total					
None	82	34.2	Referent		0.01^*^
One	87	36.3	2.36	(0.72, 7.77)	0.16
Two or more	71	29.6	5.59	(1.75, 17.81)	<0.001^*^
*Work characteristics*					
*Location*					
Ward/Pool^3^/TASU^4^/Other^5^	112	46.7	Referent		0.08
Emergency	31	12.9	0.76	(0.19, 3.12)	0.70
Intensive care	35	14.6	3.87	(1.23, 12.12)	0.02^*^
Theatre/Outpatients/Gastro^6^/DPC^7^	62	25.8	2.05	(0.71, 5.95)	0.18
*Average hours worked per week*					
<36	111	46.3	Referent		
> = 36	129	53.8	2.28	(0.91, 5.71)	0.08

^1^Body mass index categories (WHO guidelines: underweight/normal (<24.99), overweight (25–29.99), obese (>30.00).

^2^SF36 (Acute Version) Physical Component Summary Scale.

^3^Nursing Pool – Nurses allocated to different work areas each shift to cover workload demands.

^4^Theatre Admission Supply Unit.

^5^Other – includes administration, Central Sterilising.

^6^Gastroenterology outpatients.

^7^Day procedures centre.

^*^statistically significant.

## Discussion

MSDs of the foot/ankle were relatively common, being reported by more than 40% of nurses during the past seven days and more than 50% during the past 12 months in our survey conducted among paediatric hospital nurses in Brisbane, Australia. These prevalences are comparable to complaints reported for other body regions, including the lower back, which have historically received more attention in the scientific, clinical and occupational health literature. Moreover, almost 20% of nurses described their problems as activity limiting, suggesting adverse consequences for the workplace and home life. Among the nurse and work characteristics analyzed, obesity, physical health in the lowest quartile, two or more foot conditions, and working in the ICU appear to be independent risk factors, each associated with a four-fold or greater increased odds of reporting foot/ankle MSDs that limit the nurses’ physical activity. These are discussed below.

Obesity and overweight is more prevalent in nurses than in the general populations in Australia, the United Kingdom and New Zealand but the reasons for this are not well understood [21]. Obesity has been associated with an increased risk of work-related musculoskeletal injuries [22] and foot [23], lower-limb and lower-back pain [24,25]. Obesity alters gait [26-28], causing the foot to pronate [28] and increases pressures exerted beneath the foot, especially among women [29]. Few studies have investigated the effects of weight loss on foot disorders but there is some evidence that weight loss can reduce general foot pain [23].

In our study, those nurses with the poorest physical health were six times more likely to have experienced foot/ankle problems limiting their activity. Analysis of the open-ended survey comments from these nurses revealed that some were working with significant illnesses, such as osteoarthritis, rheumatoid arthritis and autoimmune diseases such as systemic lupus erythematosis, each of which can directly affect the feet [30]. In a study of registered nurses in the United States (n =1171) 62% of nurses reported that health problems had affected their work productivity to some extent and musculoskeletal pain was their most common health complaint [31]. It has been predicted that with the ageing of the nursing workforce there will be more nurses working whilst suffering from health problems and more will need to be done to support these nurses to remain in the workforce [32].

The foot conditions such as bunions (hallux valgus), toe deformities, flat feet, high arches, corns/callous and heel spurs/problems have all been associated with foot pain [33], however, the finding that there was a more than five-fold increase in the likelihood of reporting a disabling foot/ankle MSD for nurses with two or more of these conditions provides some insight into their impact. Interestingly, nurses who used insoles/orthoses were more likely to report disabling foot conditions, which may indicate these nurses were seeking treatment for a condition they had identified. Given that these conditions can often be ameliorated with interventions such as appropriate modifications to footwear and use of foot orthoses, we recommend the provision of targeted education for nurses regarding self-management strategies and treatment options, such as podiatry treatment.

The single most important work factor to be associated with activity-limiting foot/ankle MSDs was working in the ICU. The almost four-fold increase in these problems amongst ICU nurses may have been related to the nature of the work tasks or potentially the shift duration, as this was the only hospital work unit to use 12 hour shifts at the time of the study. Trinkoff et al. [34] demonstrated an increase in musculoskeletal disorders of the neck, back and shoulders in their longitudinal study of nurses commencing shifts of 13 hours or longer. This appeared to be related to the increase in physical stress, as the study adjusted for psychological demands and evidence of the detrimental effects of 12 hour shifts on nurses’ health and quality of patient care appears to be growing [35]. Theatre nurses in one study identified prolonged standing and walking (up to 10 hrs) as the main contributing factor to foot/ankle MSDs in their work location [9]. Further research is required into the relationship between 12 hour shifts and foot MSDs, as well as the physical demands of ICU work tasks.

### Strengths and limitations

Although cross-sectional in nature, this study has provided important information regarding the high prevalence of foot/ankle MSDs among nurses and the fact that both individual characteristics of nurses and the nature and location of their work are linked to the occurrence of foot/ankle MSDs that are disabling. The sample reflected the broader nursing workforce in terms of distribution across gender, nursing positions and full-time vs part-time positions. However, it should be noted that the study targeted hospital nurses engaged in work tasks caring for paediatric patients, which may differ somewhat from the broader nursing workforce. A number of issues may have conservatively biased the prevalence estimates, namely, the younger (mean) age of the nurses in this study, the hospital admissions and nursing workload were at their lowest for the year (based on bed days and admissions) and nurses may have under-reported their MSDs, as observed in other studies [36,37]. There were some additional limitations to the study. Psychosocial factors, such as job stress were not considered as risk factors in our study. Grouping of nurses across work locations also may have affected results, although this was done in consultation with nursing staff in an attempt to ensure that workload characteristics in pooled categories were as similar as possible. The study did not determine if nurses electing to use insoles/orthoses initiated this as self-treatment or if they had been prescribed by health professionals. Further research is required to determine not only the effectiveness of orthoses, but also the extent to which other interventions e.g. medication are being utilised by nurses to relieve foot problems. Measurement of actual time periods nurses spent on their feet would have been a more accurate measure than hours worked per week (study measure), however it would have involved a more resource intensive observational design or relied on self-report.

## Conclusions

This study is the first to specifically investigate the prevalence of foot/ankle MSDs and the risk factors for disabling foot/ankle MSDs in a group of paediatric hospital nurses; a group previously under-represented in the literature for prevalence of MSDs. Health issues of obesity, poorer physical health and having multiple underlying foot conditions were associated with a four-fold to six-fold increase in the likelihood of nurses experiencing disabling foot/ankle MSDs. Working in ICU on shifts of 12 hours or longer was the single work factor found to independently increase the odds of experiencing these foot problems. We recommend nurses be provided with targeted education regarding self-care strategies and treatment options for managing foot problems and more research be undertaken into the physical demands on the lower limb when working in the ICU or working 12 hour shifts.

## Competing interests

The authors declare that they have no competing interests.

## Authors’ contributions

Design and concept: LFR, DB, JY, BN. Data collection: LFR, JY. Data processing and statistical analysis: LFR, DB, BN. Interpretation of findings: LFR, DB, JY, BN. LFR wrote the first version of the manuscript. All authors took part in reading and editing the paper and approving the final version of the manuscript.

## Pre-publication history

The pre-publication history for this paper can be accessed here:

http://www.biomedcentral.com/1471-2474/15/196/prepub
